# Metformin Improves Quality of Post-Thaw Canine Semen

**DOI:** 10.3390/ani10020287

**Published:** 2020-02-12

**Authors:** Jérémy Grandhaye, Agnieszka Partyka, Zuzanna Ligocka, Agata Dudek, Wojciech Niżański, Eric Jeanpierre, Anthony Estienne, Pascal Froment

**Affiliations:** 1 INRAE UMR85 Physiologie de la Reproduction et des Comportements, 37380 Nouzilly, France; jeremy.grandhaye@inrae.fr (J.G.); eric.jeanpierre@inrae.fr (E.J.); anthony.estienne@inrae.fr (A.E.); 2 CNRS UMR7247 Physiologie de la Reproduction et des Comportements, 37380 Nouzilly, France; 3 Université François Rabelais de Tours F-37041 Tours, France; 4 IFCE, 37380 Nouzilly, France; 5 Faculty of Veterinary Medicine, Department of Reproduction and Clinic of Farm Animals, Wroclaw University of Environmental and Life Sciences, Pl. Grunwaldzki 49, 50-366 Wroclaw, Poland; zuzanna.ligocka@gmail.com (Z.L.); agata.dudek@gmail.com (A.D.); wojciech.nizanski@upwr.edu.pl (W.N.)

**Keywords:** metformin, canine sperm, cryopreservation, mitochondria, AMPK

## Abstract

**Simple Summary:**

Cryopreservation of semen is getting easier, however, fertilizing results after insemination with frozen-thawed semen is still not constant in canine species depending on the breed and could be still improved. In this study, we decided to modulate the mitochondrial activity through the addition of metformin in semen extender to increase germ cells’ quality. Metformin presented the absence of toxicity and an improvement in sperm motility after thawing, as well as an increase in the expression of several molecular markers associated with quality. In addition, the oxidative stress and DNA damage were reduced in semen frozen in the presence of metformin. Overall, these data suggest that metformin added in canine semen extender has beneficial effects on canine semen quality and could be associated with different components such as vitamins, to enhance the antioxidants status.

**Abstract:**

Sperm cryopreservation is an assisted reproductive technique routinely used in canine species for genetic conservation. However, during cryopreservation, the DNA damages are still elevated, limiting the fertilization rate. The present study was conducted to evaluate whether supplementation of canine semen extender with a molecule limiting the metabolic activities can improve the quality of frozen-thawed canine spermatozoa. We used metformin, known to limit the mitochondrial respiratory and limit the oxidative stress. Before and during the freezing procedure, metformin (50 µM and 500 µM) has been added to the extender. After thawing, sperm exposed to metformin conserved the same viability without alteration in the membrane integrity or acrosome reaction. Interestingly, 50 µM metformin improved the sperm motility in comparison to the control, subsequently increasing mitochondrial activity and NAD^+^ content. In addition, the oxidative stress level was reduced in sperm treated with metformin improving the sperm quality as measured by a different molecular marker. In conclusion, we have shown that metformin is able to improve the quality of frozen-thawed dog semen when it is used during the cryopreservative procedure.

## 1. Introduction

Cryopreservation of semen from several species is increasingly used for genetic conservation or to preserve the genetic reserve of endangered species. The cryopreservative medium is adapted to the physiology of the species and the semen capacities (membrane quality, motility, oxidative stress, DNA preservation, and fertilization). Hence, the canine semen present some specificity as low resistance to cooling as sperm from swine [1,2], or to be sensitive to fructose [3,4], and to present a membrane composition with high cholesterol: phospholipid ratio, and rich in polyunsaturated fatty acids which increase the sensitivity to lipid peroxidation [5,6]. Despite cryopreservation of semen is getting easier, fertilizing results after insemination with frozen-thawed semen are still not constant even in mammals and especially with feline or canine species [7,8,9,10]. Many factors can potentially influence reproductive success after artificial insemination. One of them is the oxidative stress leading to membrane perturbation and DNA damage. The aim of the extenders is to protect the semen against ice crystal formation, but also to improve the membrane and DNA quality in order to increase the success of fertilization [11,12]. The cryopreservative extender frequently used in veterinary practice could still be optimized to limit the plasma membrane degradation and DNA damage during chilled storage [13,14]. Indeed, several studies have reported that abnormal sperm morphology is associated with lipid peroxidation (LPO) and antioxidant imbalance, caused by the occurrence of oxidative stress [15]. Oxidative stress is a common phenomenon in spermatozoa, induced by stress such as hypoxia or temperature changes, leading to excessive production of reactive oxygen species (ROS) and oxidation of spermatozoon components [16,17,18,19,20]. To counteract these effects, different antioxidant families such as plant extracts, enzymes, and vitamins are available but have some limits alone [21,22,23]. 

In the present study, we have focused on metformin, molecule modulating the cell metabolism through the mitochondria activity as tested in extender for the cryopreservation of mouse [24], chicken [25], stallion [26] or boar [27] sperm. In mouse, metformin is allowed to increase the rate of in vitro fertilization and reduced the number of abnormal zygotes, showing a potential interest. Metformin, is a synthetic molecule from the biguanide family, commonly used for the treatment of type II diabetes and which has been described to possess an antioxidant capacity [28,29]. Metformin has the ability to decrease reactive oxygen species [30] and to activate the transcription factor Nrf2, inducing an increase in the expression of antioxidant genes [31,32]. In addition, the mechanism of action of metformin involved partly the AMP-activated protein kinase (AMPK) kinase in somatic cells [33,34], which is a key regulator of cellular energy balance, and also activated in species with a natural freezing tolerance or during hibernation [35]. 

The aim of the present study was to evaluate the effect of the supplementation of canine semen extender with metformin on the viability, motility, oxidative stress and markers of sperm quality by using in vitro analysis.

## 2. Materials and Methods 

### 2.1. Animals and Semen Collection

Six Slovakian Hound stud dogs ranging between 2 and 6 years of age from a private kennel were used in this study. The young adult dogs were patients of the Department of Reproduction and Clinic of Farm Animals in Wroclaw University. The animals were in good health and normal reproductive condition and were maintained in the kennel and fed dry food once daily, with free access to water. The semen from each dog was collected by manual stimulation once a week for seven consecutive weeks. The sperm-rich fraction of each ejaculate was collected into a calibrated collection tube with water-coat pre-warmed to 37 °C. Semen was collected as part of a routine reproductive examination of dogs. There were no associated ethical issues in this clinical study and all the dog breeders involved gave written consent for their participation, as they saw the study as vital to future breeding programs of the breed.

### 2.2. Cryopreservation and Thawing Method

The Tris-citric acid–fructose–egg yolk extender (TFE) is composed of Tris (hydroxymethyl)-aminomethane (0.2 M), citric acid monohydrate (0.06 M), fructose (0.05 M), distilled water, and with 20% (v/v) addition of egg yolk [36,37]. Each week, each ejaculate was analyzed to determine its semen concentration, the total number of spermatozoa and sperm motility, to obtain an adequate semen quality. After these analyses, all ejaculates were pooled. Then, the pool of sperm was divided into three aliquots in order to evaluate 3 different cryopreservative diluents—1/metformin 50 µM, 2/metformin 500 µM, 3/control. Each aliquot was centrifuged at 500 g for 5 min at room temperature. The pellet was resuspended to give the final concentration of 100 × 10^6^ spermatozoa per milliliter. 

Sample A was resuspended in TFE supplemented to 50 µM of metformin, sample B in TFE with 500 µM of metformin, and sample C in the same extender alone (control). Each extended semen was cooled to 5 °C over 1 h then 6% (final concentration) of glycerol was added. Samples were equilibrated for 90 min at 5 °C then cryopreserved [36,37]. On the day of the analysis, the samples were thawed at 37 °C in a water bath for 60 s, then experiments were performed.

The relevant concentration of metformin to inhibit the mitochondrial respiratory chain complex 1 in various cell types is <100 μM and the metformin levels measured in treated patients with diabetes mellitus are about (40–70 µM) [38]. Thus, the 50 µM metformin concentration was chosen to obtain an optimal activity without adverse effects as already described in germ cells [39,40].

### 2.3. Computer-Assisted Semen Analysis

Sperm motility was evaluated using computer-assisted semen analyzer (CASA) Hamilton-Thorne Sperm Analyser IVOS version 12.2l (Hamilton Thorne Biosciences, MA, USA) under 1.89 × 10 magnification. Three µl aliquot of semen was placed in a Leja analysis chamber (Leja, Nieuw-Vannep, Netherlands) at 37 °C. Five fields were randomly selected for analysis. The parameters measured were: the percentage of motile sperm (MOT), the percentage of progressively motile spermatozoa (PMOT), path velocity (VAP, average velocity/smoothed average position of the spermatozoa), progressive velocity (VSL, straight line distance between the beginning and the end of the track), curvilinear line velocity (VCL, average velocity measured over the actual point-to-point track followed by the cell), straightness (STR, a measure of VCL side to side movement determined by the ratio VSL/VAP × 100), linearity (LIN, a measure of the departure of the cell track from a straight line; the ratio VSL/VCL × 100), and percentage of rapid spermatozoa (RAPID).

### 2.4. Criteria of Semen Quality Analysed by Flow Cytometry 

Flow cytometric analyses were performed on a Guava EasyCyte 5 cytometer (Merck KGaA, Darmstadt, Germany). The fluorescent probes used in the experiment were excited by an Argon ion 488 nm laser. Acquisitions were done using the GuavaSoft™ 3.1.1 software (Merck KGaA, Darmstadt, Germany). The non-sperm events were gated out based on scatter properties and not analyzed. A total of 40,000 events were analyzed for each sample.

### 2.5. Sperm Membrane Integrity

Sperm membrane integrity was determined by a double-fluorescent labeling technique, according to the protocol described by Partyka et al. [41]. Briefly, 300 µL of the diluted samples were stained with 5 µL of SYBR-14 (commercial solution diluted 50-fold) and 5 µL of 1.4 mM PI (propidium iodide) (Invitrogen™, Eugene, Oregon, USA). The PI negative and SYBR-14 positive population showing green fluorescence was considered alive, with the sperm plasma membrane intact (PMI).

### 2.6. Mitochondrial Activity

Sperm mitochondrial activity was determined using staining with the JC-1 and PI (Invitrogen™, Eugene, Oregon, USA). From each sample, 500 µL of a sperm solution containing 50 × 10^6^ cell/mL was stained with 0.67 µL of 3mM JC-1 stock solution. The samples were incubated at 37 °C in the dark for 20 min before flow cytometric analysis [42]. Sperm emitting orange fluorescence was classified with a high mitochondrial membrane potential (HMMP), and those emitting only green fluorescence as low mitochondrial activity.

### 2.7. Oxidative Stress Analysis

The oxidative stress was detected by using a fluorogenic probe to measure reactive oxygen species (ROS) in live cells. Just after thawing, the spermatozoa were incubated with CellRox (CellRox, Invitrogen, Cergy-Pontoise, France) at a final concentration of 5 μM and was incubated for 30 min at 37 °C. Cells were washed with PBS and observed by fluorescence microscopy (Zeiss Axioplan 2; Zeiss Gruppe, Jena, Germany). Quantification of the staining intensity was estimated by using the software ImageJ (NIH, Bethesda, Maryland, USA) on at least 500 spermatozoa per animal (3 animals per condition).

Lipid peroxidation was evaluated using a fluorescent lipid probe C11-BODIPY^581/591^ 4, 4-difluoro-5-(4-phenyl-1,3-butadienyl)-4-bora-3a,4a-diaza-s-indacene-3-undecanoic acid; (Invitrogen™, Eugene, Oregon, USA) as we described before [43] and analyzed by flow cytometer. The color of the probe is changed according to the peroxidation state (non-peroxidized: red; peroxidized: green). Thawing semen was centrifuged to remove egg yolk, then samples were diluted with Tris-citric acid–fructose extender to concentration 50 × 10^6^ cell/mL and incubated with 1 μL of 2 mM C11-BODIPY^581/591^ in ethanol for 30 min at 37 °C in the dark. Samples were centrifuged at 500 g for 3 min and the sperm pellets were re-suspended in 500 μL of Tris-citric acid–fructose extender (TF). Detection of green fluorescence was set with a FL-1 band-pass filter (530/30 nm) and red fluorescence was measured using a FL-3 long-pass filter (620 nm).

### 2.8. Energetic Metabolites Analysis

Ten million thawed spermatozoa were resuspended in *Ca^2+^* and *Mg^2+^* free *PBS* and cells were disrupted by three cycles of freezing (liquid nitrogen)-thawing (ice). Then, the concentration of ATP, NAD^+^, and HDAC activity was obtained by the following commercial kits (Cell-Titer-Glo^TM^ Assay, NAD/NADH-Glo^TM^ Assay, HDAC-Glo I/II^Tm^ Assay kits, Promega, Madison, USA). The luminescence was quantified by using an Ascent Luminoskan luminometer (ThermoScientific, Palm Beach, FL, USA).

Concentrations of lactate were obtained by enzymatic assay (Lactate Assay Kit, Sigma-Aldrich, Saint-Louis, USA). The measurements were carried out according to the manufacturer’s protocol.

Localization of molecular markers by immunochemistry. After thawing, the spermatozoa were fixed with PAF 4% for 15 min at room temperature and were deposited on a slide. To saturate the aldehyde groups, the slides were incubated with PBS 1X/0.1 M glycine for 15 min, then cells were permeabilized with 0.1% Triton X-100 (w/v) in PBS for 15 min, and nonspecific binding sites were blocked in 2% BSA for 15 min. Cells were incubated for 60 min at room temperature with the following primary antibody: monoclonal mouse antibodies against DNA damage (Sigma-Aldrich, l’Isle d’Abeau Chesnes, France), polyclonal rabbit antibodies against hsp70, phospho-AMPKα (Thr172), phospho-p44/42 MAPK (Erk1/2) (Thr202/Tyr204), Phospho-(Ser/Thr) PKA Substrate, Acetyl-Histone H3 (Lys9, Lys14) purchased from Cell Signaling. Mouse or rabbit IgG was purchased (Sigma-Aldrich, l’Isle d’Abeau Chesnes, France) and were used as the negative control. All primary antibodies are diluted at 1:100 in 1% BSA/PBS. After the first antibody incubation, spermatozoa were washed three times in PBS. Then, spermatozoa were incubated for 45 min at room temperature with a second fluorescent antibody (goat anti-rabbit or anti-mouse IgG Alexa Fluor^®^ 488 antibodies, diluted at 1:500 in 1% BSA/PBS). Cells were counterstained with 4′, 6′-diamidino-2-phenylindole (DAPI), mounted on glass slides with Fluoroshield mounting medium (Sigma-Aldrich, l’Isle d’Abeau Chesnes, France) and examined using standard immunofluorescence microscopy. Quantification of the staining intensity was estimated by using the software ImageJ (NIH, Bethesda, Maryland, USA) on at least 500 spermatozoa per animal (3 animals per condition).

### 2.9. Statistical Analysis 

Data from the CASA system and flow cytometric assessments were arcsine transformed. One-way ANOVA followed by Newman-Keuls test by using GraphPad Prism 6 (La Jolla, CA, USA) was used to evaluate differences between groups for sperm quality parameters or biochemical parameters in frozen-thawed spermatozoa. The results are expressed as mean ± SEM. Values were determined to be significant when * *p* < 0.05, ** *p* < 0.01, *** *p* < 0.001; or by different letters indicating significant difference between groups (*p* < 0.05).

## 3. Results

### 3.1. Viability and Motility

The percentage of viable spermatozoa observed by Sybr/PI staining (Table 1) was similar in all conditions of the freezing procedure. In addition, the intact acrosomes of canine spermatozoa were not altered whatever the exposure in comparison to frozen control (Table 1). No change in ATP or lactate content was noted whatever the group (Supplementary data 1A and B).

The frozen and thawed sperm incubated with metformin (50 µM, 500 µM) during cryopreservation has presented more motile sperm (MOT) with higher progressive motility (PMOT): +1.6-fold (*p* < 0.05) and an elevated rapidity (Table 2). The straightness (VSL/VAP) was significantly lower (2%) after cryopreservation with metformin 500 µM, suggesting that sperm were not lazily moving along, but confirm that they were quite rapid (Table 2). 

**Figure 1:**
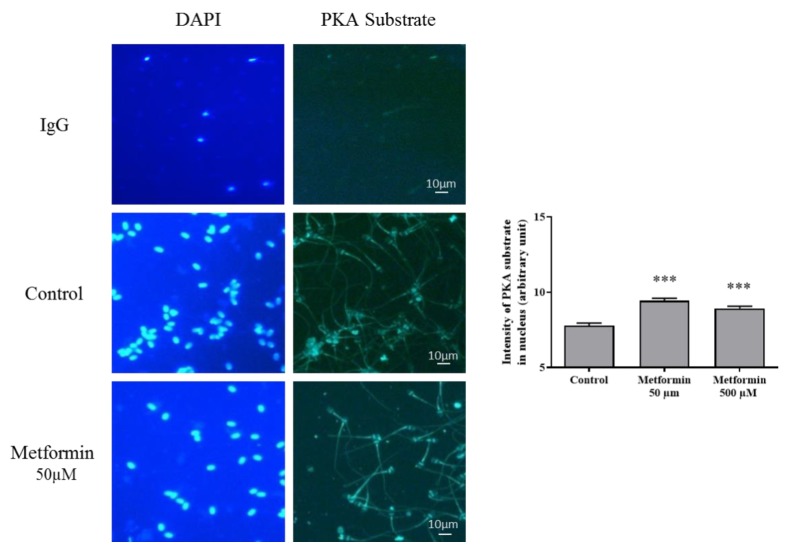
Effect of metformin on Phospho-(Ser/Thr)-PKA substrate in frozen-thawed canine semen. Immunohistochemical staining against Phospho-(Ser/Thr)-PKA substrate was assessed in frozen-thawed canine semen treated with metformin (Scale bar = 10 µm). Sperm nucleus was stained with 4′, 6′-diamidino-2-phenylindole (DAPI). Quantification of the intensity of Phospho-(Ser/Thr)-PKA substrate staining in the sperm head is represented as mean ±SEM ((arbitrary units), (n = 4 animals, with at least 500 spermatozoa per sample). *** *p* < 0.001, significant differences with the control.

The immunostaining of the phospho-(Ser/Thr) PKA Substrate, which is associated with sperm motility, was more intensely stained in both metformin groups compared to control (*p* < 0.001) (Figure 1). In addition, the high motility observed in the group “metformin 50 µM” was associated with a 1.3-fold increase in the high mitochondrial membrane potential (HMMP) (*p* < 0.05) (Table 3).

**Figure 2:**
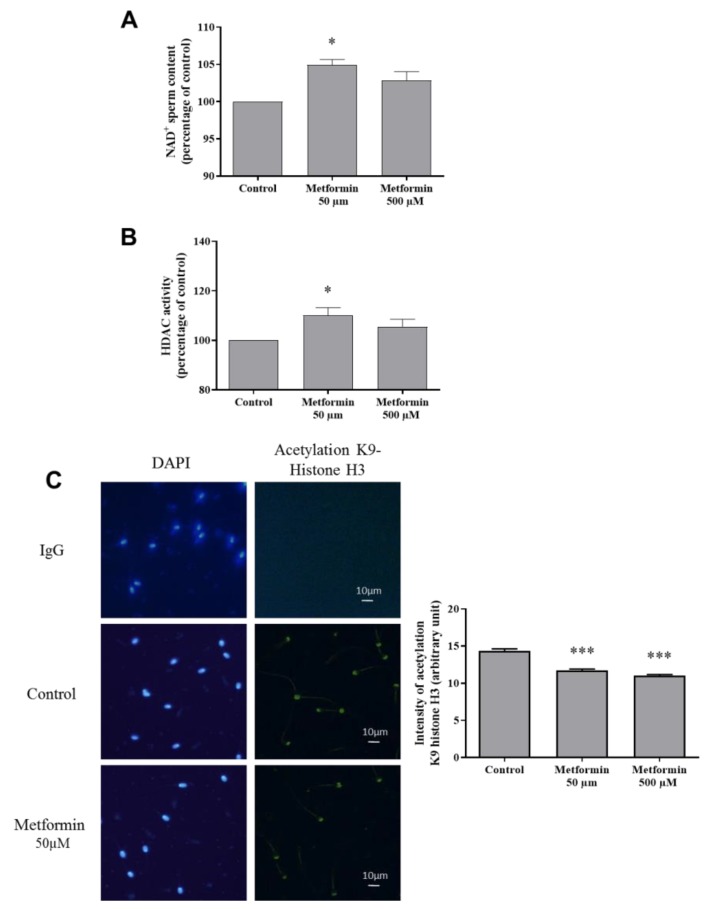
Effect of metformin on NAD^+^ content and histone deacetylase enzyme (HDAC) activity in frozen-thawed canine semen. (**A**). Intracellular NAD^+^ spermatozoa content (nM per million spermatozoa). Values are normalized as a percentage of control and represented as mean ±SEM, (n = 4). (**B**) Measurement of NAD^+^-dependent HDAC activity. (**C**) Immunostaining against a target of HDAC, the histone H3K9ac (Scale bar =10 µm). Sperm nucleus was stained with DAPI. Quantification of the H3K9ac staining intensity (arbitrary units) is represented as mean ± SEM, (n = 3 animals, with at least 500 spermatozoa per sample). * *p* < 0.05, ** *p* < 0.01, *** *p* < 0.001, significant differences with the control.

To complete the mitochondrial activity analysis, we have noted an increase in the NAD^+^ content, a metabolic coenzyme/co-substrate, playing a key role in mitochondrial function, when sperm was frozen with metformin (50 µM) (*p* < 0.05) (Figure 2A). Because NAD^+^ serves as a substrate for the histone deacetylase enzyme (HDAC), we have analyzed the HDAC activity which was higher in the metformin (50 µM) group in comparison to control (Figure 2B). A consequence reported, is a lower acetylation staining due to the deacetylase activity of the HDAC enzyme in spermatozoa from the metformin groups (Figure 2C).

### 3.2. Molecular Markers Associated to Semen Quality

**Figure 3:**
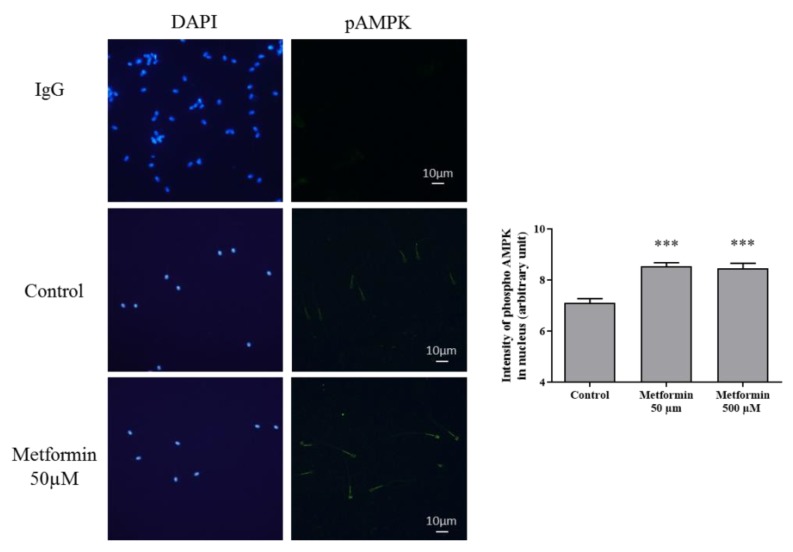
Effect of metformin on AMPK phosphorylation in frozen-thawed canine semen. Immunostaining against phospho-AMPK was assessed in frozen-thawed canine semen treated with metformin (Scale bar = 10 µm). Sperm nucleus was stained with DAPI. Quantification of the intensity of phospho-AMPK staining in the sperm head is represented as mean ±SEM (arbitrary units), (n = 4 animals, with at least 500 spermatozoa per sample). *** *p* < 0.001, significant differences with the control.

To determine the mechanism of action of metformin, we immunostained the phospho-AMPK protein, which is activated by metformin or in case of ATP depletion. The phospho-AMPK protein was localized in the sperm head and in the mid-piece (Figure 3). The staining of phospho-AMPK increased after metformin (50 µM, 500 µM) exposure in comparison to the control condition (Figure 3; *p* < 0.01).

**Figure 4:**
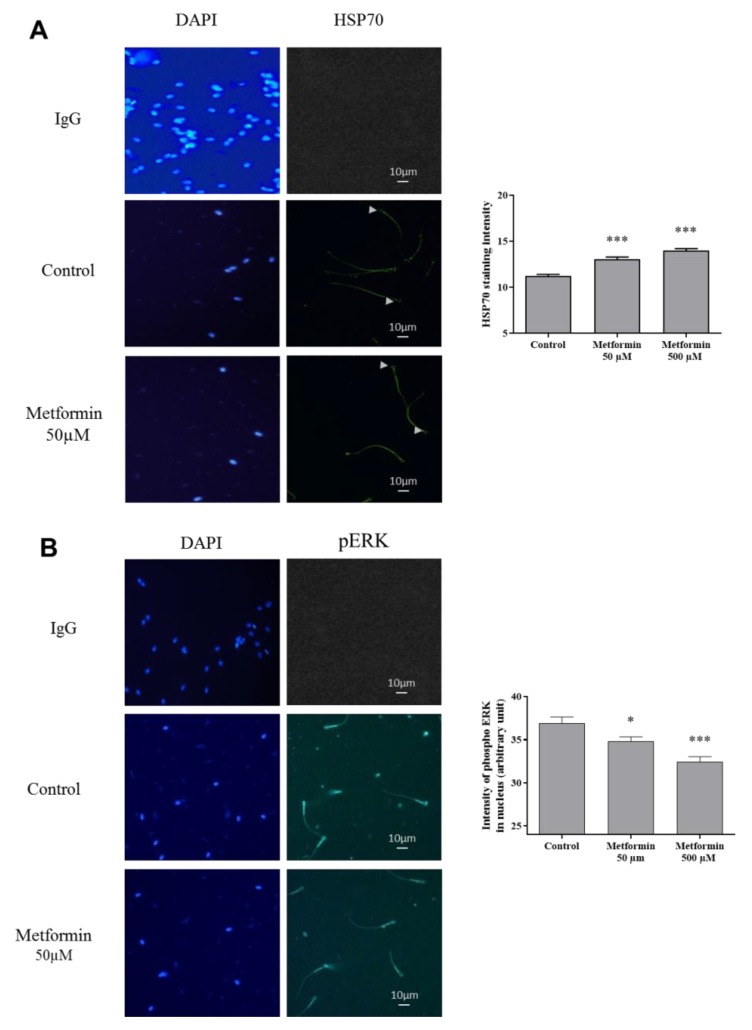
Effect of metformin on markers associated to quality in frozen-thawed canine semen. (**A**) Immunostaining against HSP70, and (**B**) phospho-ERK was assessed in frozen-thawed canine semen (Scale bar = 10 µm). Sperm nucleus was stained with DAPI. Quantification of the intensity of HSP70, and phospho-ERK staining in the sperm head (arbitrary units) are represented as mean ± SEM, (n = 4 animals, with at least 500 spermatozoa per sample). * *p* < 0.05, ** *p* < 0.01, *** *p* <0.001, significant differences with the control.

Analysis of hsp70, a marker of sperm quality, located in the equatorial region of the sperm head, was stronger expressed in sperm treated with metformin (*p* < 0.001) (Figure 4A) and inversely, the phospho-ERK (localize in mid-piece and in the sperm head), which is described to be present in case of stress, is lower stained in presence of metformin (50 µM, *p* < 0.05 and 500 µM, *p* < 0.001) (Figure 4B).

### 3.3. Oxidative Stress 

**Figure 5:**
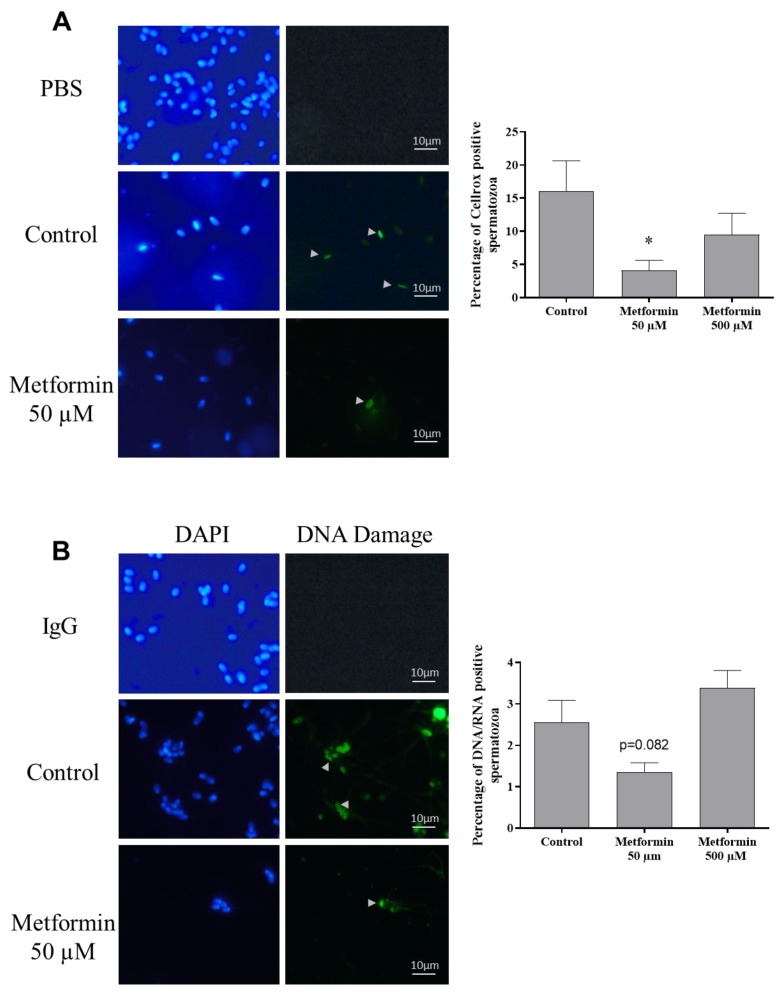
Effect of metformin on oxidative stress and DNA damage in frozen-thawed canine semen. (**A**) Oxidative stress was detected with the fluorescent CellRox probe and (**B**) immunostaining against DNA damage was assessed in frozen-thawed canine semen treated with 0, 50 µM or 500 µM metformin (Scale bar = 10 µm). PBS (**A**) and mouse IgG (**B**) incubation were used as negative controls for CellRox and immunostaining, respectively. Sperm nucleus was stained with DAPI. (**A**,**B**) Quantification of the spermatozoa labeled with CellRox or DNA damage by ImageJ. Data are represented as mean ±SEM, (n = 4 animals, with at least 500 spermatozoa per sample). * *p* < 0.05; significant differences with the control.

Oxidative stress which altered the sperm quality was evaluated through lipid peroxidation (LPO) and presence reactive oxygen species content (Cellrox staining). Hence, just after semen thawing, a strong, significant decrease in reactive oxygen species (80%) was observed when sperm was cryopreserved with metformin (50 µM) (*p* < 0.05) (Figure 5A). However, no consequence of lipid peroxidation was reported (Table 4). The decrease in oxidative stress in the metformin (50 µM) group was associated with a tendency to lower the percentage of spermatozoa stained for DNA damage (*p* = 0.082) (Figure 5B).

## 4. Discussion

The use of the classical Tris-fructose extender with egg yolk is able to cryopreserve the dog semen. However, for patients with low semen quality (dogs present more than 350 heritable disorders/traits and some with fertility) or for endangered canine species, the percentage of high semen quality is not optimal. The improvement of the semen extender to increase the semen protection and quality could be realized by limiting energy production before cryopreservation and to conserve after thawing, the energy for sperm mobility and to limit oxidative stress [13,15]. In the present study, we have analyzed the effect of metformin, which is known to limit the mitochondria activity and to induce an antioxidant barrier as observed in somatic cells [44]. The data obtained in this study has confirmed that a canine extender supplemented with metformin can improve post-thaw motility and reduced oxidative stress. 

Metformin can penetrate in spermatozoa through cell membranes passively or by transporters such as MATE1 or MATE2 which is strongly expressed in testis [45]. In different species, metformin has been reported to stimulate AMPK phosphorylation [24,46,47]. In this study, metformin was shown to stimulate AMPK in dog sperm as in stallion and chicken. Moreover, the phosphorylated AMPK was also located in the sub-equatorial band in the sperm head and in the mid-piece which concentrates the mitochondria [25,26] suggesting a link between the metformin/AMPK in the control of energy production, and motility. 

In various cell types, it has been observed a metformin activity with a lower concentration than 100 µM inhibiting notably the mitochondrial respiratory chain complex 1 [38]. In mice, the 50 µM metformin concentration is also enough to increase semen viability and to impact cell metabolism [39]. However in others species, a higher concentration was tested such as in chicken, a treatment with 1 mM metformin (20-fold more than in mouse) lead to an increase in viability and mobility [47], or in boar fresh sperm a 5 mM treatment with metformin partially reduce the motility [46]. Thus, we can suppose that the dog species present a high sensitivity to metformin in comparison to other species, and the use of higher metformin concentration is not so relevant [40].

In addition, we have observed that metformin did not alter sperm viability or the acrosome as in other publications on the mouse, stallion, chicken, and boar species [24,25,26,46]. Thus, we confirm the absence of toxicity of metformin in our conditions by PI staining and caspase 3 activity which is known to be a marker of cell death [48]. Moreover, we have observed beneficial effects on oxidative stress. It is known that the freezing and thawing process exacerbated the oxidative stress and altered the membrane, cell organelles, such as mitochondria and DNA [49]. In our conditions, the use of metformin in canine sperm have been allowed to decrease the oxidative stress and limit the percentage of spermatozoa with endamaged DNA. Semen with a low oxidative stress status and low DNA damage have been showed to improve the future embryo quality and development [50]. The antioxidant ability of metformin has been described recently in somatic or germ cells with an elevated metformin concentration (2 mM) in comparison to physiological conditions (10–50 µM). In rooster, the authors have shown that metformin increased the activity of antioxidant enzymes to decrease ROS content in frozen-thawed rooster sperm [47]. We can suggest in canine cryopreserved semen, that adding metformin in extenders can slow down oxidative stress and DNA fragmentation, which could limit effects on sperm chromatin integrity and improve the embryo quality [51].

We have shown in the presence of metformin that the motility of canine sperm was increased and was associated with a hyper mitochondrial activity as measured by mitochondrial membrane potential and higher NAD^+^ production. We can note that motility is one of the main parameters of spermatozoa known to contribute to successful fertilization and easily measurable [52,53]. NAD^+^ is a metabolite that is linked with the improvement in mitochondrial function under stress in somatic cells [54]. NAD^+^ levels decline with mitochondrial dysfunction or mitochondrial disorders [55]. In mouse spermatozoa, Bertoldo et al. have already observed that metformin during the cryopreservation process improves the mitochondria activity in thawed semen as in canine semen. The authors reported also a 5-fold increase in AMPK phosphorylation associated with an increase in the histone deacetylase (HDAC) activity in mouse spermatozoa [24]. Moreover, several recent studies have shown that metformin through AMPK activation remodeled cytoskeleton dynamics [56]. We can hypothesize that metformin/AMPK phosphorylated protein associated with the microtubules in flagella such as A kinase-anchoring protein (AKAP), a PKA substrate implicated in a flagellar beating.

The heat shock protein 70 (hsp70), a heat shock protein ensures properly protein folding to fulfill their physiological functions, has been focused by several teams working on the goat, bull, boar, and fish semen and used it as a marker of good semen quality [57,58,59,60,61,62,63,64]. Hsp70 has been described to possess a protective effect on various types of injuries and some activities involved the Sirtuin/AMPK pathway [65]. In goat semen, the cryopreservation process altered ultra-structure in sperm organelles and decreased Hsp70 expression. However, the addition of an antioxidant formulation in semen extender improved the post-thaw semen quality and increased the hsp70 levels [66]. Similarly, a model of transgenic mice deficient for hsp70-2 presented a higher level of the germ cell apoptosis [67]. Conversely, in bull and boar, hsp70 increased longevity and viability of spermatozoa [68]. In our conditions, the high hsp70 levels in spermatozoa treated with metformin in comparison to control, presume a non-degraded semen quality. We can suppose that the stimulation of the AMPK pathway is involved. Indeed, in rat intestine cells, the activation of AMPK by L-Arginine and AICAR upregulated the expression level of HSP70 and HSP90 [69].

The ERK protein is also a second marker associated with quality as described in stallion or human. Authors have shown that stallion semen with elevated quality and low oxidative stress presented lower ERK phosphorylation after semen cryopreservation [59]. In humans, a degraded semen quality has a higher ERK1 expression reinforcing the interest to use it as a predictive marker of sperm quality [57]. Despite the role of ERK in sperm motility, its activity presents some controversial effects depending on the species. Thus, a positive role for ERK was suggested in chicken sperm [70], but ERK seems to inhibit the motility of human spermatozoa [71]. In our study, the low levels of phosphorylated ERK in cryopreserved semen with metformin suggest an increase in semen quality as reported above with the other quality markers.

## 5. Conclusions

In conclusion, our results have supported evidence that the quality of canine semen cryopreserved in an extender supplemented with metformin improved its quality (motility, oxidative stress, quality markers). In addition, a mechanism of action of metformin involved the AMPK pathway in canine spermatozoa. AMPK is considered as a part of the key node regulating energy production, cytoskeleton and flagella motility, oxidative stress and DNA quality. All these parameters improved the sperm quality during stress such as in the freeze/thaw process. Finally, these data open to future experiments and to evaluate the interest to supplement the extender with metformin by using specific functional fertilizing in vitro or in vivo tests.

## Figures and Tables

**Table 1:** Effect of metformin on semen viability and acrosome reaction on frozen-thawed and fresh semen.

Group	Fresh Semen	Control	Metformin 50 µM	Metformin 500 µM
Viable	80.83 ± 1.47	49.78 ± 5.4	48.63 ± 4.8	46.67 ± 8.0
Live intact acrosomes	79.69 ± 2.8	50.19 ± 3.7	50.45 ± 3.6	50.71 ± 4.3

Data are represented as mean ± SEM (n = 6).

**Table 2:** Effect of metformin on sperm motility in fresh and frozen-thawed canine semen.

Group	Fresh Semen	Control	Metformin 50 µM	Metformin 500 µM
MOT (%)	94.00 ± 2.1	40.63 ± 4.5 ^a^	57.03 ± 5.3 ^b^	56.44 ± 7.0 ^a,b^
PMOT (%)	69.00 ± 1.5	19.08 ± 3.2 ^a^	30.42 ± 2.9 ^b^	29.82 ± 3.7 ^b^
RAPID (%)	75.33 ± 1.8	21.40 ± 3.9 ^a^	34.39 ± 3.3 ^b^	33.89 ± 4.1 ^a,b^
VAP (µm/s)	151.83 ± 5.1	117.45 ± 7.4	130.24 ± 4.0	128.83 ± 2.6
VSL (µm/s)	139.47 ± 5.4	104.57 ± 6.3	114.43 ± 4.4	112.90 ± 3.4
VCL (µm/s)	191.90 ± 4.3	168.31 ± 13.5	187.55 ± 6.6	188.52 ± 6.0
STR (%)	90.67 ± 0.3	88.20 ± 1.2 ^a^	86.44 ± 0.9 ^a,b^	86.33 ± 1.1 ^b^
LIN (%)	73.33 ± 0.9	64.64 ± 2.7	62.36 ± 2.7	61.38 ± 2.7

Characteristics measured are: percentage of motile sperm (MOT); percentage of progressively motile spermatozoa (PMOT), percentage of rapid spermatozoa (RAPID); (VAP): Average path velocity; (VSL): Straight-line velocity; (VCL): Curvilinear velocity; (STR): Straightness index; (LIN): Linearity index. Data are represented as mean ±SEM (n = 6). ^a,b^ different superscripts within the same row indicate significant differences (*p* < 0.05).

**Table 3:** Effect of metformin on mitochondria activity in fresh and frozen-thawed canine semen.

Group	Fresh Semen	Control	Metformin 50 µM	Metformin 500 µM
High mitochondrial membrane potential (HMMP)	80.27 ± 6.2	48.87 ± 6.4 ^a^	61.92 ± 8.6 ^b^	60.29 ± 10.4 ^a,b^

Data are represented as mean ±SEM (n = 6). Mitochondrial activity was determined by JC-1 staining and was represented as a percentage of high mitochondrial membrane potential. Data are represented as mean ±SEM, (n = 6). ^a,b^ different superscripts within the same row indicate significant differences (*p* < 0.05).

**Table 4:** Effect of metformin on lipid peroxidation in fresh and frozen-thawed canine semen.

Group	Fresh Semen	Control	Metformin 50 µM	Metformin 500 µM
Live with LPO	5.3 ± 1.4	1.84 ± 0.3	1.32 ± 0.2	1.61 ± 0.3

Data are represented as mean ±SEM (n = 6). Lipid peroxidation (LPO) was evaluated with the C_11_-BODIPY^581/591^ probe as described in material and methods. Lipid peroxidation was assessed in frozen-thawed canine semen treated with 50 µM or 500 µM metformin. Data are represented as mean ±SEM, (n = 6).

## Supplementary Materials

The following are available online at https://www.mdpi.com/2076-2615/10/2/287/s1.

## References

[1] Hori T., Yoshikuni R., Kobayashi M., Kawakami E. (2014). Effects of Storage Temperature and Semen Extender on Stored Canine Semen. J. Vet. Med. Sci..

[2] Pinto C.R.F., Paccamonti D.L., Eilts B.E. (1999). Fertility in bitches artificially inseminated with extended, chilled semen. Theriogenology.

[3] Ponglowhapan S., Essén-Gustavsson B., Linde Forsberg C. (2004). Influence of glucose and fructose in the extender during long-term storage of chilled canine semen. Theriogenology.

[4] Bucci D., Rodriguez-Gil J.E., Vallorani C., Spinaci M., Galeati G., Tamanini C. (2011). GLUTs and mammalian sperm metabolism. J. Androl..

[5] Drobnis E.Z., Crowe L.M., Berger T., Anchordoguy T.J., Overstreet J.W., Crowe J.H. (1993). Cold shock damage is due to lipid phase transitions in cell membranes: A demonstration using sperm as a model. J. Exp. Zool..

[6] Bencharif D., Amirat L., Anton M., Schmitt E., Desherces S., Delhomme G., Langlois M.-L., Barrière P., Larrat M., Tainturier D. (2008). The advantages of LDL (low density lipoproteins) in the cryopreservation of canine semen. Theriogenology.

[7] Hollinshead F.K., Hanlon D.W. (2017). Factors affecting the reproductive performance of bitches: A prospective cohort study involving 1203 inseminations with fresh and frozen semen. Theriogenology.

[8] Linde-Forsberg C. (1991). Achieving canine pregnancy by using frozen or chilled extended semen. Vet. Clin. N. Am. Small Anim. Pract..

[9] Niżański W., Klimowicz M., Partyka A., Savić M., Dubiel A. (2009). Effects of the Inclusion of Equex STM into Tris-Based Extender on the Motility of Dog Spermatozoa Incubated at 5 °C. Reprod. Domest. Anim..

[10] Okano T., Murase T., Asano M., Tsubota T. (2004). Effects of final dilution rate, sperm concentration and times for cooling and glycerol equilibration on post-thaw characteristics of canine spermatozoa. J. Vet. Med. Sci..

[11] Santos S.E.C., Vannucchi C.I., Satzinger S.I., Assumpcao M.E., Visintin J.A. (1999). Comparison of five extenders for canine semen freezing. Braz. J. Vet. Res. Anim. Sci..

[12] Axner E., Lagerson E. (2016). Cryopreservation of Dog Semen in a Tris Extender with 1% or 2% Soya Bean Lecithin as a Replacement of Egg Yolk. Reprod. Domest. Anim..

[13] Peña F.J., Núñez-Martínez I., Morán J.M. (2006). Semen technologies in dog breeding: An update. Reprod. Domest. Anim..

[14] Iguer-ouada M., Verstegen J.P. (2001). Long-term preservation of chilled canine semen: Effect of commercial and laboratory prepared extenders. Theriogenology.

[15] Aitken R.J., Buckingham D.W., Carreras A., Irvine D.S. (1996). Superoxide dismutase in human sperm suspensions: Relationship with cellular composition, oxidative stress, and sperm function. Free Radic. Biol. Med..

[16] Kim S.-H., Yu D.-H., Kim Y.-J. (2010). Effects of cryopreservation on phosphatidylserine translocation, intracellular hydrogen peroxide, and DNA integrity in canine sperm. Theriogenology.

[17] Kim S.-H., Yu D.-H., Kim Y.-J. (2010). Apoptosis-like change, ROS, and DNA status in cryopreserved canine sperm recovered by glass wool filtration and Percoll gradient centrifugation techniques. Anim. Reprod. Sci..

[18] Watson P.F. (2000). The causes of reduced fertility with cryopreserved semen. Anim. Reprod. Sci..

[19] Lucio C.F., Regazzi F.M., Silva L.C.G., Angrimani D.S.R., Nichi M., Vannucchi C.I. (2016). Oxidative stress at different stages of two-step semen cryopreservation procedures in dogs. Theriogenology.

[20] Sabeti P., Pourmasumi S., Rahiminia T., Akyash F., Talebi A.R. (2016). Etiologies of sperm oxidative stress. Int. J. Reprod. Biomed..

[21] Taylor K., Roberts P., Sanders K., Burton P. (2009). Effect of antioxidant supplementation of cryopreservation medium on post-thaw integrity of human spermatozoa. Reprod. Biomed. Online.

[22] Kalthur G., Raj S., Thiyagarajan A., Kumar S., Kumar P., Adiga S.K. (2011). Vitamin E supplementation in semen-freezing medium improves the motility and protects sperm from freeze-thaw-induced DNA damage. Fertil. Steril..

[23] Khodayari Naeini Z., Hassani Bafrani H., Nikzad H. (2014). Evaluation of ebselen supplementation on cryopreservation medium in human semen. Iran. J. Reprod. Med..

[24] Bertoldo M.J., Guibert E., Tartarin P., Guillory V., Froment P. (2014). Effect of metformin on the fertilizing ability of mouse spermatozoa. Cryobiology.

[25] Nguyen T.M.D., Alves S., Grasseau I., Métayer-Coustard S., Praud C., Froment P., Blesbois E. (2014). Central role of 5′-AMP-activated protein kinase in chicken sperm functions. Biol. Reprod..

[26] Córdova A., Strobel P., Vallejo A., Valenzuela P., Ulloa O., Burgos R.A., Menarim B., Rodríguez-Gil J.E., Ratto M., Ramírez-Reveco A. (2014). Use of hypometabolic TRIS extenders and high cooling rate refrigeration for cryopreservation of stallion sperm: Presence and sensitivity of 5′ AMP-activated protein kinase (AMPK). Cryobiology.

[27] Hurtado de Llera A., Martin-Hidalgo D., Gil M.C., Garcia-Marin L.J., Bragado M.J. (2015). AMPK up-activation reduces motility and regulates other functions of boar spermatozoa. Mol. Hum. Reprod..

[28] Tsuda T., Watanabe M., Ohshima K., Norinobu S., Choi S.W., Kawakishi S., Osawa T. (1994). Antioxidative Activity of the Anthocyanin Pigments Cyanidin 3-O-Beta-d-Glucoside and Cyanidin. J. Agric. Food Chem..

[29] Tsuda T., Shiga K., Ohshima K., Kawakishi S., Osawa T. (1996). Inhibition of lipid peroxidation and the active oxygen radical scavenging effect of anthocyanin pigments isolated from *Phaseolus vulgaris* L.. Biochem. Pharmacol..

[30] Esteghamati A., Eskandari D., Mirmiranpour H., Noshad S., Mousavizadeh M., Hedayati M., Nakhjavani M. (2013). Effects of metformin on markers of oxidative stress and antioxidant reserve in patients with newly diagnosed type 2 diabetes: A randomized clinical trial. Clin. Nutr..

[31] Onken B., Driscoll M. (2010). Metformin induces a dietary restriction-like state and the oxidative stress response to extend C. elegans Healthspan via AMPK, LKB1, and SKN-1. PLoS ONE.

[32] Ashabi G., Khalaj L., Khodagholi F., Goudarzvand M., Sarkaki A. (2015). Pre-treatment with metformin activates Nrf2 antioxidant pathways and inhibits inflammatory responses through induction of AMPK after transient global cerebral ischemia. Metab. Brain Dis..

[33] Chen J., Zhu Y., Zhang W., Peng X., Zhou J., Li F., Han B., Liu X., Ou Y., Yu X. (2018). Delphinidin induced protective autophagy via mTOR pathway suppression and AMPK pathway activation in HER-2 positive breast cancer cells. BMC Cancer.

[34] Zhou G., Myers R., Li Y., Chen Y., Shen X., Fenyk-Melody J., Wu M., Ventre J., Doebber T., Fujii N. (2001). Role of AMP-activated protein kinase in mechanism of metformin action. J. Clin. Investig..

[35] Horman S., Hussain N., Dilworth S.M., Storey K.B., Rider M.H. (2005). Evaluation of the role of AMP-activated protein kinase and its downstream targets in mammalian hibernation. Comp. Biochem. Physiol. B Biochem. Mol. Biol..

[36] Nizanski W., Dubiel A., Bielas W., Dejneka G.J. (2001). Effects of three cryopreservation methods and two semen extenders on the quality of dog semen after thawing. J. Reprod. Fertil. Suppl..

[37] Nizanski W. (2006). Intravaginal insemination of bitches with fresh and frozen-thawed semen with addition of prostatic fluid: Use of an infusion pipette and the Osiris catheter. Theriogenology.

[38] Foretz M., Guigas B., Viollet B. (2019). Understanding the glucoregulatory mechanisms of metformin in type 2 diabetes mellitus. Nat. Rev. Endocrinol..

[39] Bertoldo M.J., Guibert E., Faure M., Guillou F., Ramé C., Nadal-Desbarats L., Foretz M., Viollet B., Dupont J., Froment P. (2016). Specific deletion of AMP-activated protein kinase (α1AMPK) in mouse Sertoli cells modifies germ cell quality. Mol. Cell. Endocrinol..

[40] Faure M., Bertoldo M.J., Khoueiry R., Bongrani A., Brion F., Giulivi C., Dupont J., Froment P. (2018). Metformin in Reproductive Biology. Front. Endocrinol..

[41] Partyka A., Niżański W., Łukaszewicz E. (2010). Evaluation of fresh and frozen-thawed fowl semen by flow cytometry. Theriogenology.

[42] Partyka A., Nizanski W., Bajzert J., Lukaszewicz E., Ochota M. (2013). The effect of cysteine and superoxide dismutase on the quality of post-thawed chicken sperm. Cryobiology.

[43] Partyka A., Łukaszewicz E., Niżański W., Twardoń J. (2011). Detection of lipid peroxidation in frozen-thawed avian spermatozoa using C11-BODIPY581/591. Theriogenology.

[44] Ouslimani N., Peynet J., Bonnefont-Rousselot D., Thérond P., Legrand A., Beaudeux J.-L. (2005). Metformin decreases intracellular production of reactive oxygen species in aortic endothelial cells. Metab. Clin. Exp..

[45] Otsuka M., Matsumoto T., Morimoto R., Arioka S., Omote H., Moriyama Y. (2005). A human transporter protein that mediates the final excretion step for toxic organic cations. Proc. Natl. Acad. Sci. USA.

[46] Hurtado de Llera A., Martin-Hidago D., Gil M., Garcia-Marin L., Bragado M. (2012). The AMPK activator metformin inhibits one of the main functions of boar spermatozoa, motility. FEBS J..

[47] Nguyen T.M.D., Seigneurin F., Froment P., Combarnous Y., Blesbois E. (2015). The 5′-AMP-Activated Protein Kinase (AMPK) Is Involved in the Augmentation of Antioxidant Defenses in Cryopreserved Chicken Sperm. PLoS ONE.

[48] Gallardo Bolaños J.M., Balao da Silva C.M., Martín Muñoz P., Morillo Rodríguez A., Plaza Dávila M., Rodríguez-Martínez H., Aparicio I.M., Tapia J.A., Ortega Ferrusola C., Peña F.J. (2014). Phosphorylated AKT preserves stallion sperm viability and motility by inhibiting caspases 3 and 7. Reproduction.

[49] McGann L.E., Yang H.Y., Walterson M. (1988). Manifestations of cell damage after freezing and thawing. Cryobiology.

[50] Aitken R.J., Koopman P., Lewis S.E. (2004). Seeds of concern. Nature.

[51] Olaciregui M., Luño V., Gonzalez N., De Blas I., Gil L. (2015). Freeze-dried dog sperm: Dynamics of DNA integrity. Cryobiology.

[52] Amaral A., Lourenço B., Marques M., Ramalho-Santos J. (2013). Mitochondria functionality and sperm quality. Reproduction.

[53] Marchetti P., Ballot C., Jouy N., Thomas P., Marchetti C. (2012). Influence of mitochondrial membrane potential of spermatozoa on in vitro fertilisation outcome. Andrologia.

[54] Cantó C., Menzies K.J., Auwerx J. (2015). NAD+ Metabolism and the Control of Energy Homeostasis: A Balancing Act between Mitochondria and the Nucleus. Cell Metab..

[55] Srivastava S. (2016). Emerging therapeutic roles for NAD+ metabolism in mitochondrial and age-related disorders. Clin. Trans. Med..

[56] Szrejder M., Rachubik P., Rogacka D., Audzeyenka I., Rychłowski M., Kreft E., Angielski S., Piwkowska A. (2020). Metformin reduces TRPC6 expression through AMPK activation and modulates cytoskeleton dynamics in podocytes under diabetic conditions. Biochim. Biophys. Acta Mol. Basis Dis..

[57] Almog T., Lazar S., Reiss N., Etkovitz N., Milch E., Rahamim N., Dobkin-Bekman M., Rotem R., Kalina M., Ramon J. (2008). Identification of Extracellular Signal-regulated Kinase 1/2 and p38 MAPK as Regulators of Human Sperm Motility and Acrosome Reaction and as Predictors of Poor Spermatozoan Quality. J. Biol. Chem..

[58] Bai C., Kang N., Zhao J., Dai J., Gao H., Chen Y., Dong H., Huang C., Dong Q. (2019). Cryopreservation disrupts lipid rafts and heat shock proteins in yellow catfish sperm. Cryobiology.

[59] Cocchia N., Pasolini M.P., Mancini R., Petrazzuolo O., Cristofaro I., Rosapane I., Sica A., Tortora G., Lorizio R., Paraggio G. (2011). Effect of sod (superoxide dismutase) protein supplementation in semen extenders on motility, viability, acrosome status and ERK (extracellular signal-regulated kinase) protein phosphorylation of chilled stallion spermatozoa. Theriogenology.

[60] Huang S.Y., Chen M.Y., Lin E.C., Tsou H.L., Kuo Y.H., Ju C.C., Lee W.C. (2002). Effects of single nucleotide polymorphisms in the 5′-flanking region of heat shock protein 70.2 gene on semen quality in boars. Anim. Reprod. Sci..

[61] Huang S.Y., Kuo Y.H., Lee Y.P., Tsou H.L., Lin E.C., Ju C.C., Lee W.C. (2000). Association of heat shock protein 70 with semen quality in boars. Anim. Reprod. Sci..

[62] Matwee C., Kamaruddin M., Betts D.H., Basrur P.K., King W.A. (2001). The effects of antibodies to heat shock protein 70 in fertilization and embryo development. Mol. Hum. Reprod..

[63] Nikbin S., Panandam J.M., Yaakub H., Murugaiyah M., Sazili A.Q. (2014). Novel SNPs in heat shock protein 70 gene and their association with sperm quality traits of Boer goats and Boer crosses. Anim. Reprod. Sci..

[64] Spinaci M., Volpe S., Bernardini C., Ambrogi M.D., Tamanini C., Seren E., Galeati G. (2005). Immunolocalization of heat shock protein 70 (Hsp 70) in boar spermatozoa and its role during fertilization. Mol. Reprod. Dev..

[65] Liu S., Xu J., Fang C., Shi C., Zhang X., Yu B., Yin Y. (2016). Over-expression of heat shock protein 70 protects mice against lung ischemia/reperfusion injury through SIRT1/AMPK/eNOS pathway. Am. J. Transl. Res..

[66] Reddy V.S., Yadav B., Yadav C.L., Anand M., Swain D.K., Kumar D., Kritania D., Madan A.K., Kumar J., Yadav S. (2018). Effect of sericin supplementation on heat shock protein 70 (HSP70) expression, redox status and post thaw semen quality in goat. Cryobiology.

[67] Dix D.J., Allen J.W., Collins B.W., Mori C., Nakamura N., Poorman-Allen P., Goulding E.H., Eddy E.M. (1996). Targeted gene disruption of Hsp70-2 results in failed meiosis, germ cell apoptosis, and male infertility. Proc. Natl. Acad. Sci. USA.

[68] Elliott R.M.A., Lloyd R.E., Fazeli A., Sostaric E., Georgiou A.S., Satake N., Watson P.F., Holt W.V. (2009). Effects of HSPA8, an evolutionarily conserved oviductal protein, on boar and bull spermatozoa. Reproduction.

[69] Xia Z., Huang L., Yin P., Liu F., Liu Y., Zhang Z., Lin J., Zou W., Li C. (2019). L-Arginine alleviates heat stress-induced intestinal epithelial barrier damage by promoting expression of tight junction proteins via the AMPK pathway. Mol. Biol. Rep..

[70] Ashizawa K., Hashimoto K., Higashio M., Tsuzuki Y. (1997). The addition of mitogen-activated protein kinase and p34cdc2 kinase substrate peptides inhibits the flagellar motility of demembranated fowl spermatozoa. Biochem. Biophys. Res. Commun..

[71] Weidinger S., Mayerhofer A., Kunz L., Albrecht M., Sbornik M., Wunn E., Hollweck R., Ring J., Kohn F.M. (2005). Tryptase inhibits motility of human spermatozoa mainly by activation of the mitogen-activated protein kinase pathway. Hum. Reprod..

