# Supplementary material for: Metformin Improves Quality of Post-Thaw Canine Semen
Source: Animals (Basel). 2020 Feb 12;10(2):287. doi: 10.3390/ani10020287 (PMC7070956; doi:10.3390/ani10020287)
Supplement: Supplementary file 1 [file animals-10-00287-s001.pptx]

## Slide 1
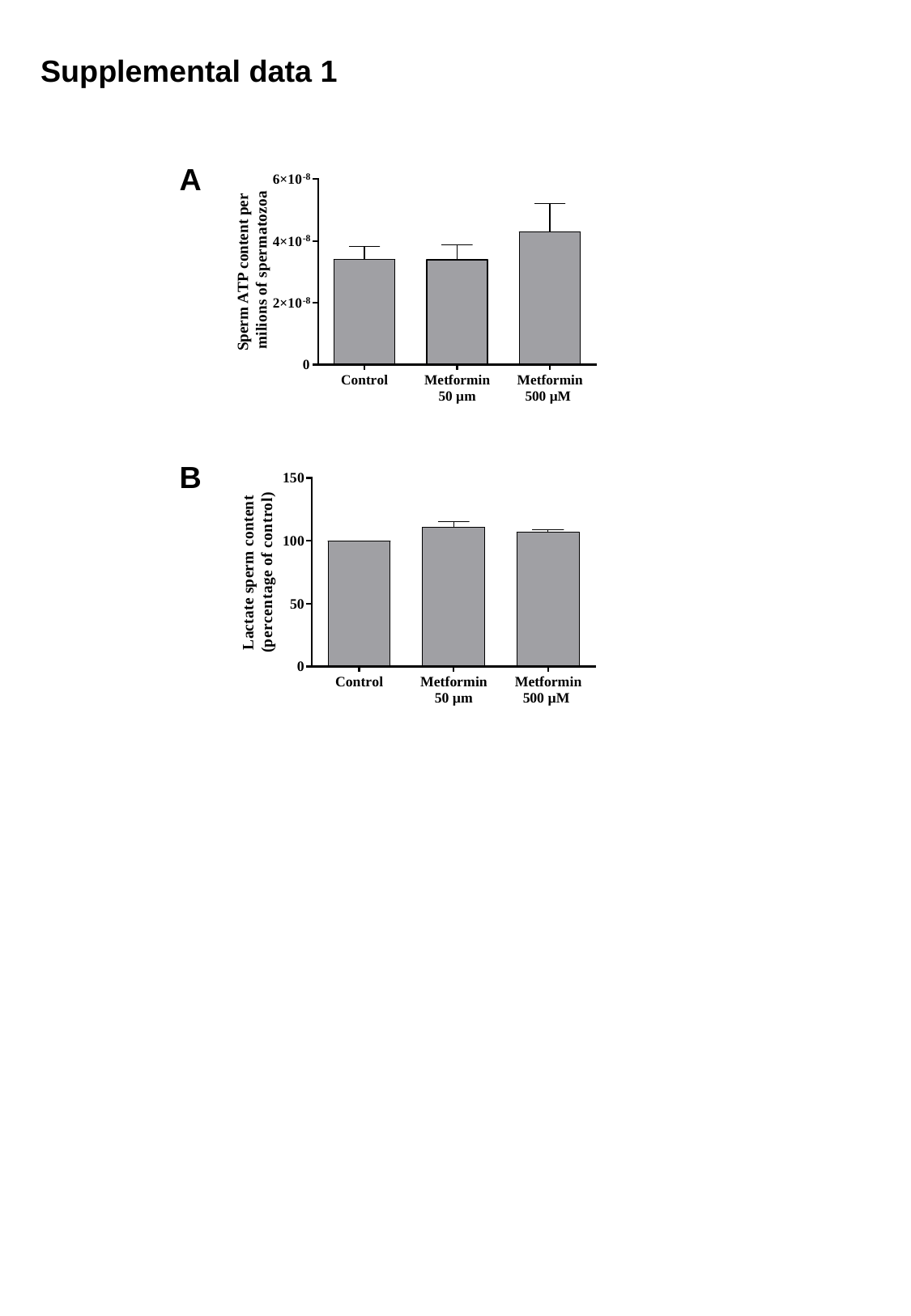

Supplemental data 1
A
B

## Slide 2
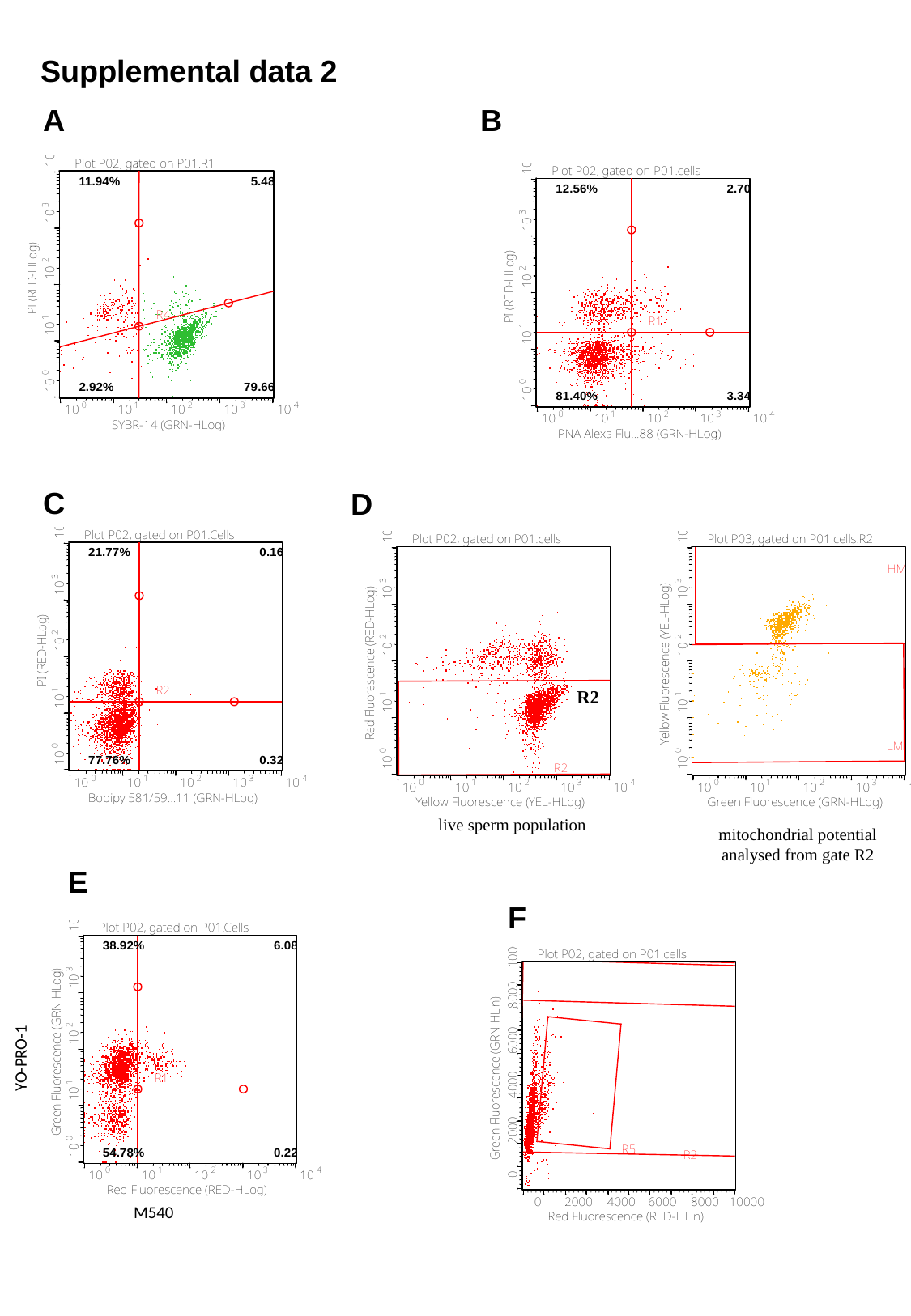

Supplemental data 2
A
B
C
D
R2
live sperm population
mitochondrial potential analysed from gate R2
E
F
YO-PRO-1
M540
